# Assessing Social Consequences for Task Completion in Individuals with Autism Spectrum Disorder

**DOI:** 10.3390/bs16091695

**Published:** 2026-09-20

**Authors:** Laura B. DeZayas, Janae’ A. Pendergrass, Arielle R. Marshall, Justin B. Han, Faris R. Kronfli, Timothy R. Vollmer

**Affiliations:** Department of Psychology, University of Florida, Gainesville, FL 32603, USA; j.pendergrass@ufl.edu (J.A.P.); marshall.arielle@ufl.edu (A.R.M.); justinhan@ufl.edu (J.B.H.); kronfli.faris@ufl.edu (F.R.K.); vollmera@ufl.edu (T.R.V.)

**Keywords:** autism, concurrent operant assessment, praise, preferred conversation topics, social preferences

## Abstract

We used a concurrent operant assessment (COA) to evaluate social preferences for individuals with autism spectrum disorder across three comparison conditions: praise versus no interaction, preferred conversation topics versus no interaction, and preferred conversation topics versus praise. First, a multiple stimulus without replacement preference assessment was conducted to identify the preferred conversation topics and interests for all participants. Second, a COA was conducted where participants were able to choose which consequence they wanted to receive while working on an academic task during each comparison condition. We found that five out of six participants allocated more time to the praise side over no interaction, five out of six participants allocated more time to the preferred conversation topics over no interaction, and four out of six participants allocated more time to the preferred conversation topics over praise. The results indicate that social stimuli such as praise and preferred conversation topics may function as preferred stimuli indicating a need for future researchers and clinicians to individualize reinforcers. Thus, a proof-of-concept study is established to evaluate procedures for indicating preferences for types of social interaction.

## 1. Introduction

Individuals with autism spectrum disorder (ASD) present with persistent difficulties with social-communication and social interaction (American Psychiatric Association, 2022). These social-communication difficulties can limit an individual’s ability to form and maintain relationships and may also hinder their ability to participate and succeed in academic, occupational, and broader community settings (Eaves & Ho, 2008; Kasari et al., 2011; Lee & Carter, 2012). One theoretical framework that may explain why individuals with ASD may face challenges with social interaction is the social motivation theory, which suggests that individuals with ASD are less sensitive to social stimuli (e.g., facial expressions and social rewards), which limits opportunities to develop social skills and maintain meaningful relationships (Chevallier et al., 2012). On the contrary, it has been demonstrated that children with autism do engage in social initiations with peers (Black et al., 2024; Kasari et al., 2011) but are unable to take part in more fulfilling social interactions (e.g., making friendships) due to a lack of social interaction skills and intersubjective understanding of social relationships (Bauminger et al., 2003). It may be due to these difficulties that children with autism report struggling with feelings of loneliness and exclusion (Bauminger et al., 2003).

Researchers have demonstrated that social interactions with peers (e.g., Yuan & Chen, 2020) and adults (e.g., Clay et al., 2013; Kelly et al., 2014; Koegel et al., 2009; Morris & Vollmer, 2021; Nuernberger et al., 2012; Smaby et al., 2007) can function as preferred stimuli and reinforcers for children with ASD. In one example, Morris and Vollmer (2021) evaluated an assessment to determine the function of social interactions for children with ASD. Their procedures included splitting a room in half with tape on the floor and placing identical sets of toys on both sides of the room with tables and chairs. Sessions started with an adult picking one side of the room to stay on (i.e., social side) and then the child was guided into the room and able to move about freely. The adult switched to the other side of the room every two minutes or when the participant manded for them to switch. They found that nine out of 21 participants allocated more time to the side of the room where an adult was located rather than the side of the room where they were alone which demonstrated that social interactions were reinforcing. Seven participants allocated similar time to the social side and alone side of the room, and five participants spent more time on the alone side of the room. Similarly, Koegel et al. (2009) assessed whether embedding social interactions into known reinforcing activities would lead to increased child-initiated social behavior for three children with ASD. They compared a non-embedded reinforcer condition with an embedded social condition. During the non-embedded reinforcer condition, children’s verbal responses were reinforced with their preferred items or activities, and the reinforcing activities were presented without embedded social interactions (e.g., an adult played a child’s preferred song and did not interact with them). During the embedded social condition, children’s verbal responses were reinforced by providing access to their preferred items or activities where social interactions by the adults were included (e.g., an adult played a child’s preferred song and sang with them). The results indicated that there were increases in self-initiated social engagement, such as orienting, and an improvement in the child’s affect when social interactions were embedded into reinforcers. These findings suggest that children with autism do, in fact, seek out social interactions with others, and such interactions may function as an effective conduit for reinforcers.

Despite a variety of social activities in clinical practice and research being reported as highly preferred and highly reinforcing, praise is the most commonly delivered social reinforcer within behavior analysis (Morris & Bridges, 2024a; Morris et al., 2024b). However, there are varied results when praise is used as a putative “reinforcer” for individuals with ASD (Senn et al., 2020). For example, Senn et al. (2020), in Study One, evaluated the effectiveness of using praise as a reinforcer during a reinforcer assessment using mastered tasks with six preschoolers aged three to five. Two participants had a diagnosis of ASD, one had a diagnosis of developmental delay, and three did not have any diagnosis. They found that praise was an effective reinforcer for one participant with ASD and one participant with a developmental delay. Praise was not found to be an effective reinforcer for the other four participants. In Study Two, they examined whether praise would function as a reinforcer for a novel task with two of the participants from Study One where praise was a reinforcer and two participants where praise was not found to be an effective reinforcer. Their results showed that praise was not an effective reinforcer for any participants and prompts may have been a controlling variable for the acquisition of skills. Jimenez-Gomez et al. (2024) also found variable effectiveness of using praise statements as a reinforcer. In Experiment One, they examined whether a novel praise statement paired with edibles would enhance the resistance to extinction of a mastered motor imitation response following response stimulus pairing. The results of Study One demonstrated that praise statements enhanced the resistance to extinction and maintained high levels of responding for a mastered task. In Experiment Two, they examined whether the same paired praise statements presented as a consequence could be used to teach a novel response. The results demonstrated that responding was higher when edibles were delivered than when the praise statements were delivered. This further suggests that, because praise is presumably a conditioned reinforcer, praise may not be an effective reinforcer for all individuals with ASD, given that its efficacy as a reinforcer may depend on the history of pairing with other conditioned or unconditioned reinforcers (Stahl et al., 1974). Additionally, reinforcement schedules, the task being completed, and context may also impact the effectiveness of praise statements (Jimenez-Gomez et al., 2024; Kranak et al., 2017). Similarly, individuals with ASD who present with lower cognitive scores or have deficits in communication skills such as listener repertoire skills may not understand the semantic meaning of praise statements. These skill deficits may impact the efficacy of praise statements delivered by adults (Gale et al., 2023). Collectively, the findings highlight the need to individualize reinforcer selection, as some social stimuli, such as praise, may not be the most potent, despite its ubiquitous usage.

Although social reinforcers such as praise or attention are a commonly implemented consequence in service settings, the selection of social reinforcers is not always individualized (Morris & Bridges, 2024a). Researchers within behavior analysis have developed several methods to assess the preference for social interactions in individuals with ASD, including using social interaction preference assessments (e.g., Morris & Vollmer, 2019; Morris et al., 2023), paired-stimulus preference assessments (e.g., Clay et al., 2018; Kelly et al., 2014), video-based preference assessments (e.g., Wolfe et al., 2018), graphics interchange format (GIF)-based preference assessments (e.g., Morris & Vollmer, 2020), concurrent operant chains (e.g., Gutierrez et al., 2013; Lloyd et al., 2019), response restriction conversation assessments (e.g., Kronfli et al., 2023), and picture-based multiple stimulus without replacement preference assessments (MSWO) (e.g., DeLeon & Iwata, 1996; Kang et al., 2013; Nuernberger et al., 2012). These diverse experimental arrangements have helped clarify whether social stimuli function as reinforcers and provide a basis for the development of procedures to individualize social stimuli used for individuals with ASD.

There is emerging evidence that preferred conversation topics may function as reinforcers for individuals with ASD (Fisher et al., 2013; Stocco et al., 2021). For example, Stocco et al. (2021) evaluated a social skills training procedure where a researcher would provide differential attention for on-topic speech. Specifically, if the participant engaged in a conversation about the researcher’s target topic, the researcher would then act as an interested listener. If the participant engaged in off-topic speech, the researcher would act as an uninterested listener. In the next condition, they conducted a contingency reversal where researchers responded as interested listeners following off-topic speech and as uninterested listeners during on-topic speech. During the final condition, the researcher provided differential attention for on-topic speech and then allowed the participants to talk about their own preferred conversation topics. Study Two assessed participants’ preferences for the intervention features. The results of Study Two demonstrated that most participants showed a preference for the intervention that involved contingent access to preferred conversation topics. In another study, Clay et al. (2018) conducted preference assessments within and across edible, physical, and vocal (preferred conversation topics) stimulus classes for two participants diagnosed with ASD who were aged 5 and 24. Although, for both participants, the preference was highest for edible relative to physical or vocal reinforcers (preferred conversational topics), preferred conversational topics functioned as reinforcers for both participants and maintained higher response rates relative to a control condition.

Social stimuli could be useful, as they are easy to deliver, inexpensive, and presumably have fewer long-term side effects compared to, say, edible reinforcers or screen viewing (e.g., Chen et al., 2016; Kang et al., 2013; Kemp et al., 2024; Must et al., 2014). Additionally, praise and conversations may be more ecologically valid as they are easily delivered by different people across different contexts and are common in human interactions. Further, it is possible that practitioners overuse praise as a reinforcer (e.g., continuously saying “good job!”). For example, Vollmer and Iwata (1991) showed that, even though praise was a reinforcer for one participant, it lost its effect and even became aversive when presented too frequently. Therefore, it is important that researchers continue to assess the preference for and reinforcer effectiveness of social stimuli such as preferred conversation topics for individuals with ASD. Research within neuroscience has also demonstrated that social learning and social interactions lead to positive brain changes as well as enhanced behavioral outcomes (Li & Jeong, 2020). To contribute to the growing body of literature on social preference, the purpose of the current study was to use a COA to evaluate whether six individuals with ASD are (a) allocated responding toward praise or no interaction, (b) allocated responding toward preferred conversation topics or no interaction, and (c) allocated responding toward preferred conversation topics or praise. A secondary aim was to assess the number of tasks completed during each condition of the COA. We did not formulate any hypotheses before data collection due to the analysis being exploratory. Apart from the potential utility and contribution to the literature on social reinforcement, this study serves as a proof-of-concept aimed at evaluating an approach to identify preferences for different types of social interactions.

## 2. Methods

Five children and one adult with ASD were recruited to participate. Parental and/or participant consent was obtained prior to the start of the study and participant assent was obtained prior to the start of the study and before each session. August was a 19-year-old male with a diagnosis of ASD who attended college. He did not wish to disclose his race or ethnicity. August displayed limited vocal verbal behavior. He had difficulty expanding his statements and maintaining on-topic conversation. Sam was a 10-year-old White and Hispanic female with a diagnosis of ASD level 1, anxiety, Sensory Processing Disorder, and Attention Deficit/Hyperactive Disorder (ADHD). She was in the 5th grade and had advanced vocal verbal behavior where she was able to answer an array of questions and communicate using complete sentences. Matt was a 7-year-old White and Hispanic male who had a diagnosis of ASD level 2 and ADHD. He was in the 2nd grade. He had advanced vocal verbal behavior where he communicated using complete sentences and was able to answer novel questions. Eric was a 7-year-old White male with a diagnosis of ASD level 1 and ADHD. He was in the 2nd grade and had advanced vocal verbal behavior where he communicated using complete sentences, answered questions, and asked questions. Milo was a 9-year-old White male with a diagnosis of ASD and KBG syndrome. Milo was in the 3rd grade and had limited vocal verbal skills where he used a limited number of complete sentences to communicate and had difficulty answering novel questions and responding to novel statements. Eddy was a 13-year-old mixed-race male with a diagnosis of ASD. He was in the 5th grade and had limited vocal verbal communication where he would occasionally not respond to questions or statements from others. Eddy would also regularly repeat scripts from media or sing songs.

### 2.1. Materials and Settings

The study was conducted within a research clinic for five participants and in an applied behavior analysis (ABA) clinic for one participant (Milo). Sessions in the research clinic were conducted in a room (2.69 m × 3.02 m) with a table (1.47 m × 0.61 m) and four chairs. Two chairs were placed on either side of the table facing each other. The researchers placed two sets of identical tasks on the sides of the table associated with each experimental condition (e.g., no interaction vs. praise). The room contained a camera which was mounted on the wall near the ceiling and wall decorations (e.g., photographs). Sessions for Milo were conducted in a therapy space with two conjoined rooms. Each room was approximately 2.13 m × 2.13 m. The rooms contained wall decorations, desks, chairs, and storage cubbies. Milo’s sessions were conducted on a table (0.74 m × 0.60 m) with four chairs, two facing each other at either end of the table. The researchers worked with the participants’ caregivers to select mastered academic tasks for inclusion in the COA sessions. Participants used pencils and crayons to complete worksheets. Researchers held a magazine, book, or sheets with crossword puzzles to signal they were “busy” on the no interaction side. Data were collected by the researchers using the Countee application (Peic-Gavran & Hernández Eslava, 2020) on their cellular phones. A handheld recorder and wall-mounted recorder were also used to record sessions for participants whose parents gave consent for audiovisual recording. Researchers in this study consisted of the first, second, and third authors and undergraduate research assistants.

### 2.2. Measurement

#### 2.2.1. Dependent Variables

The dependent variable was the percentage of time on a side corresponding to the praise or preferred conversation topic, or no interaction side, which was defined as the participant sitting down on the chair corresponding to a particular consequence. Researchers calculated percentage of session time on one side by dividing the duration the participant was sitting on a specific side by the total session duration. Researchers also recorded the total number of task responses completed on each chosen side. Task completion for tangram puzzles included measuring the frequency of each completed puzzle. Task completion for worksheets included measuring the frequency of each completed question. Task completion for matching sheets included measuring the frequency of correctly matching one visual to the corresponding visual on the sheet. Task completion for tracing included measuring the frequency of every short word or singular letter on the worksheet that was traced.

#### 2.2.2. Social Validity

The authors developed a social validity interview form (see Table 1) and administered it after the completion of the study to assess participants’ perceptions of the study procedures. The measure consisted of a brief, structured questionnaire with seven questions. The questionnaire was developed by the authors to capture participant’s acceptability of and satisfaction with the procedures used in this specific study. Researcher-developed questionnaires about intervention-specific questions are typical in behavior analysis due to the individualized nature of the procedures (Park & Blair, 2019). The specific questions developed for this study were derived from prior studies that solicited social validation from direct participants (see Lerman et al., 2013) and were adapted to meet the communication levels of the current participants (e.g., using smiley face options for all participants except August). Prior research has also demonstrated the use of Likert questions and open-ended questions to assess social validity with participants (Gomes et al., 2025; Sureshkumar & Zonneveld, 2024). Participants first rated their overall experience using a scale ranging from “I really didn’t like it” to “I really liked it” with each option containing corresponding visuals. For example, the option “I really liked it” contained a green smiley face. For August, the first question was rated using a Likert scale ranging from one (extremely dislike) to seven (extremely like). Open-ended questions probed what aspects of the study each participant liked or disliked. The interview also included three questions evaluating their preferences in each comparison condition, to determine whether their verbal response would match their choices during sessions. Finally, participants indicated whether they would choose to participate in the study again by selecting “Yes,” “Maybe,” or “No,” accompanied by a corresponding visual. For August, a Likert scale of one (very unlikely) to seven (very likely) was used for the last question.

#### 2.2.3. Interobserver Agreement

Interobserver agreement (IOA) on percentage of time on one side (no interaction, praise, or preferred conversation topics) was assessed by a second observer for 33% of all sessions for each participant. IOA was calculated using the proportional method (Morris & Vollmer, 2021), by dividing the session into 10-second intervals and comparing each observer’s data interval by interval. The smaller duration was divided by the larger duration which corresponded to each comparison condition. The quotients were summed, divided by the total number of intervals, and multiplied by 100 to yield a percentage. However, if both observers scored 0, that interval was considered to have 100% agreement.

The proportional method was also used to calculate IOA for the frequency of completed tasks during each session for 33% of sessions within each comparison, with one exception. For Milo, 13–17% of sessions within each comparison condition had IOA for task completion. IOA for task completion was calculated similar to the proportional method described above, except that the smaller and larger number of responses (rather than duration) was evaluated for each 10-second bin. Otherwise, the IOA calculation was the same as described above.

IOA on the percentage of time sitting on the chair corresponding to the praise or no interaction side across all participants was 99% (range: 85–100%). IOA on the percentage of time sitting on the chair corresponding to the preferred conversation topic or no interaction side across all participants was 99% (range: 87–100%). IOA on the percentage of time sitting on the chair corresponding to the praise or preferred topic side was 99% (range: 94–100%).

IOA on task completion corresponding to the praise or no interaction side across all participants was 88.7% (range 69–100%). IOA on the average percentage of task completion corresponding to the preferred topic or no interaction side across all participants was 91.45% (range 62–100%). IOA on the average percentage of task completion corresponding to the praise or preferred topic side across all participants was 89.15% (range 62–100%).

IOA was also collected on the duration of praise and preferred conversation topic statements from researchers during the COA sessions. Data was collected from video recordings for at least 33% of sessions across comparison conditions for participants. Data was not collected for Sam and Matt because their caregiver did not consent to video or audio recording. IOA was calculated using the proportional method. IOA scores averaged 99.6% for Eric, 98.3% for Milo, 100% for Eddy, and 97% for August.

### 2.3. Procedural Integrity

Procedural integrity data were collected for researchers implementing the MSWO assessment, adhering to the pre-exposure script, and delivering the programmed consequences (no interaction, preferred conversation topics, or praise) during sessions. For Sam and Matt, integrity data were recorded in vivo by undergraduate research assistants. For August, Eddy, Milo, and Eric, integrity data were scored from video recordings by the first author and undergraduate research assistants. Procedural integrity corresponding data tables are provided in the Supplementary Materials. Integrity for conducting the MSWO assessment and following the pre-exposure script was reported as the mean percentage of opportunities in which the researcher engaged in the correct step. The number of correct responses was divided by the total number of steps multiplied by 100 to yield a percentage. Procedural integrity was also calculated for delivering the programmed consequence while the participant was on the corresponding side. Integrity was calculated by splitting the session into 30-second intervals and collecting data on whether only the predetermined consequences were delivered within the interval. The number of correct intervals was divided by the total number of intervals and multiplied by 100 to yield a percentage. The percentage of sessions during which procedural integrity measures were collected varied across participants. Mean procedural integrity scores for conducting the MSWO assessment ranged from 93.3% to 100%. Mean procedural integrity scores for adhering to the pre-exposure script ranged from 80% to 100%. Mean procedural integrity scores for delivering only the predetermined consequences during the COA sessions were 100%.

### 2.4. Procedures

The researchers first conducted a brief interview with caregivers and the participants to identify the participants’ preferred conversation topics. Caregivers were also asked to identify tasks that were within each participant’s skill repertoire and for which they would require minimal assistance to complete independently. Items were selected such that they produced a permanent product (e.g., written worksheet responses).

### 2.5. Multiple Stimulus Without Replacement Preference Assessment

An MSWO preference assessment similar to the one described by Kronfli et al. (2023) was conducted to determine a ranking of most preferred to least preferred conversation topics. One assessment was conducted at the beginning of every visit. The researcher placed individual index cards with the pre-identified topics written on them horizontally on the table. If the participant was unable to read, a corresponding picture was added to the index card. Participants were asked, “which one is your favorite?” The participant could choose their preferred topic by naming the card or physically touching or pointing to it. Next, they were shuffled and re-presented except for the card chosen prior. This was repeated until all topics were selected. In the interest of efficiency, we did not provide access to conversation topics after they were chosen. Table 2 provides the conversation topics identified for each participant. Topics are listed in order of most preferred to least preferred.

### 2.6. Concurrent Operant Assessment

#### 2.6.1. Comparison Conditions

Three comparison conditions were assessed using a COA. The comparison conditions included praise versus no interaction, preferred conversation topics versus no interaction, and preferred conversation topics versus praise. The order of comparisons was interspersed across sessions, and the researcher assigned to each comparison was randomized. Visits occurred one to three times per week for each participant. The number of sessions conducted varied per participant with a range of three to six sessions per visit. Sessions occurred in succession, although participants could take a break at any time.

#### 2.6.2. In-Session Tasks

For Matt and Sam, tangram puzzles were selected as the in-session task. For August and Eddy, worksheets (reading and spelling, respectively) were completed during sessions. Eric worked on tangram puzzles for the first three sessions across comparison conditions and then worked on reading comprehension worksheets for the remainder of the sessions. This change was made due to Eric completing the puzzles very quickly. Task completion for tangram puzzles included measuring the frequency of how many puzzle pieces were put on the table to complete the picture. For Milo, matching sheets such as matching colors, numbers, and letters were used in the first sessions, but, later, this was changed to tracing worksheets. This change was due to Milo having difficulty with matching independently for some pages but not others. Consequences such as praise or preferred conversation topics were delivered on a fixed ratio (FR) 1 schedule contingent on task completion as indicated above for worksheets, matching, and puzzles.

#### 2.6.3. Pre-Exposure Sessions

During the pre-exposure session, the participant was briefly exposed to each consequence corresponding to the comparison condition. For example, if the praise vs. preferred conversation topics comparison was being implemented, the participant was brought to the room and prompted to stand equidistant from both chairs at the table. The lead researcher then said “If you do your work with [researcher A’s name], they will tell you what a good job you are doing and if you do your work with [researcher B’s name] they will talk to you about dinosaurs, movies, and sharks. Let’s try both of them.” The lead researcher then prompted the participant to sit with each researcher (researcher A and B) and complete three to five responses, while receiving the predetermined consequence on an FR1 schedule. For August (first phase), Matt, and Sam, two different researchers were seated at both ends of the table with two chairs facing them on opposite sides. There were also two identical tasks placed on both sides of the table in front of the researchers. For August (second phase), Eric, Eddy, and Milo, only one researcher delivered both consequences. For these participants, the researcher would stand in the middle of the table and sit down in the chair in front of the participant. When the participant was guided to the other side of the table, the researcher followed them. This change was made to mitigate bias toward any particular researcher. Additionally, for Eddy and Milo, instead of two identical tasks on the table, one task was placed in the middle of the table and was moved by the lead researcher to be in front of the participants, wherever they were seated. This change was made due to observing previous participants taking the same worksheet packet they were working on to whichever side of the table they switched to or avoiding doing the same tasks again. Pre-exposure sessions were conducted before every COA throughout the study. The side associated with each consequence was randomized across sessions for all participants.

#### 2.6.4. Concurrent Operant Assessment

Following the pre-exposure sessions, the participant was guided to stand equidistant from both work areas, and the lead researcher said, “Just a reminder, if you sit here (while pointing to one side) researcher ‘A’ will [consequence was inserted] and if you sit here (while pointing to one side) researcher ‘B’ will [consequence was inserted]. Where do you want to sit? If you want to switch sides at any time, you may.” The lead researcher delivered this statement across the three comparison conditions (praise vs. no interaction, preferred conversation topics vs. no interaction, and praise vs. preferred conversation topics). The participant was then able to select a side of the table to sit while receiving the corresponding consequence on an FR1 schedule contingent on completing one response in the task (e.g., completing one problem on the spelling worksheet). Sessions for August, Sam, and Matt were five minutes. Sessions for Eric, Milo, and Eddy were two minutes per caregiver’s requests to increase efficiency. The lead researcher who provided the instructions remained in the room during sessions and stood one to two meters away from the participant. Criteria for participant preference for a consequence within each condition was determined by an overall higher percentage of time allocated toward a specific consequence across sessions within a condition. Termination of sessions was determined by visual analysis of the data or due to scheduling conflicts with the participant’s caregivers.

In the praise arrangement, the researcher provided brief praise contingent on response completion (e.g., “Wow! That’s looking great! Keep it up!”). In the no interaction arrangement, the researcher engaged in a task to signal they were “busy” (e.g., reading a magazine, or doing a crossword puzzle) and unable to interact with the participant. In the preferred conversation topics arrangement, the researcher either initiated a statement or asked a question about one or more of the participant’s preferred conversation topics determined from the MSWO preference assessment (e.g., “I heard you like to collect DVDs. I have so many at home!”). If the participant initiated a statement first after the completion of one task, the researcher would respond with an on-topic statement or question. If the participants engaged in verbal behavior relating to their preferred conversation topics before completing one response, the researcher kept a neutral expression and did not respond until the one response was completed. If the participants continuously attempted to engage in a verbal interaction with the researcher, the researcher would deliver a prompt such as “Let’s do the next one so we can keep talking.” If the participant completed the task at a high rate, the researcher would deliver the corresponding consequence nearly continuously (or as close to that as possible).

#### 2.6.5. Researcher Vocalizations

Researcher-delivered praise statements or verbal behavior relating to preferred conversation topics was not equalized and varied across participants. This was an unplanned difference. Due to this, a secondary variable measured was the average duration of praise or preferred topic statements/questions delivered by the researchers on the praise or preferred topic side per response. First, second-by-second data of praise statements or preferred topic statements/questions were coded from video recordings of sessions for four out of six participants. Two participants’ caregivers did not consent to video recordings, and, therefore, the secondary analysis was not conducted for them. Praise statements were defined as any instance of the researcher delivering a verbal statement of approval directed toward the participant. Praise statement included a mix of general and descriptive praise statements. Examples included the researcher saying “Wow, I’m really impressed at how fast you are completing these” or “This is looking really good!” Preferred conversation topic statements and questions were defined as any instance of the researcher delivering verbal statements or questions related to the participant’s identified preferred conversation topic. Examples included “What is your favorite Mario game?” and “I saw the Mario movie last month.” The average total duration of researcher-delivered praise statements or preferred topic statements and questions across each session was divided by the frequency of completed tasks per session while on the preferred topic or praise side. The quotient was then equal to the average duration of preferred topic statements/questions delivered by the researchers per one response while on the preferred topic side and the average duration of praise statements delivered by the researchers per one response while on the praise side.

A table of the data are depicted in Table 3. For August, across each comparison, the average amount of time the researcher delivered praise statements contingent on one response while on the praise side was 3.8 s. The average amount of time the researcher engaged in preferred conversation topics contingent on one response while on the preferred conversation topics side was 8.9 s. For Eric, across each comparison, the average amount of time the researcher delivered praise statements contingent on one response while on the praise side was 2.3 s. The average amount of time the researcher engaged in preferred conversation topics contingent on one response while on the preferred conversation topics side was 5.3 s. For Milo, across each comparison, the average amount of time the researcher delivered praise statements contingent on one response while on the praise side was 2.0 s. The average amount of time the researcher engaged in preferred conversation topics contingent on one response while on the preferred conversation topics side was 3.6 s. For Eddy, across each comparison, the average amount of time the researcher delivered in praise statements contingent on one response while on the praise side was 2.4 s. The average amount of time the researcher engaged in preferred conversation topics contingent on one response while on the preferred conversation topics side was 4.5 s. This unintended difference will be presented as a limitation and direction for future research in the discussion section.

## 3. Results

**Figure 1:**
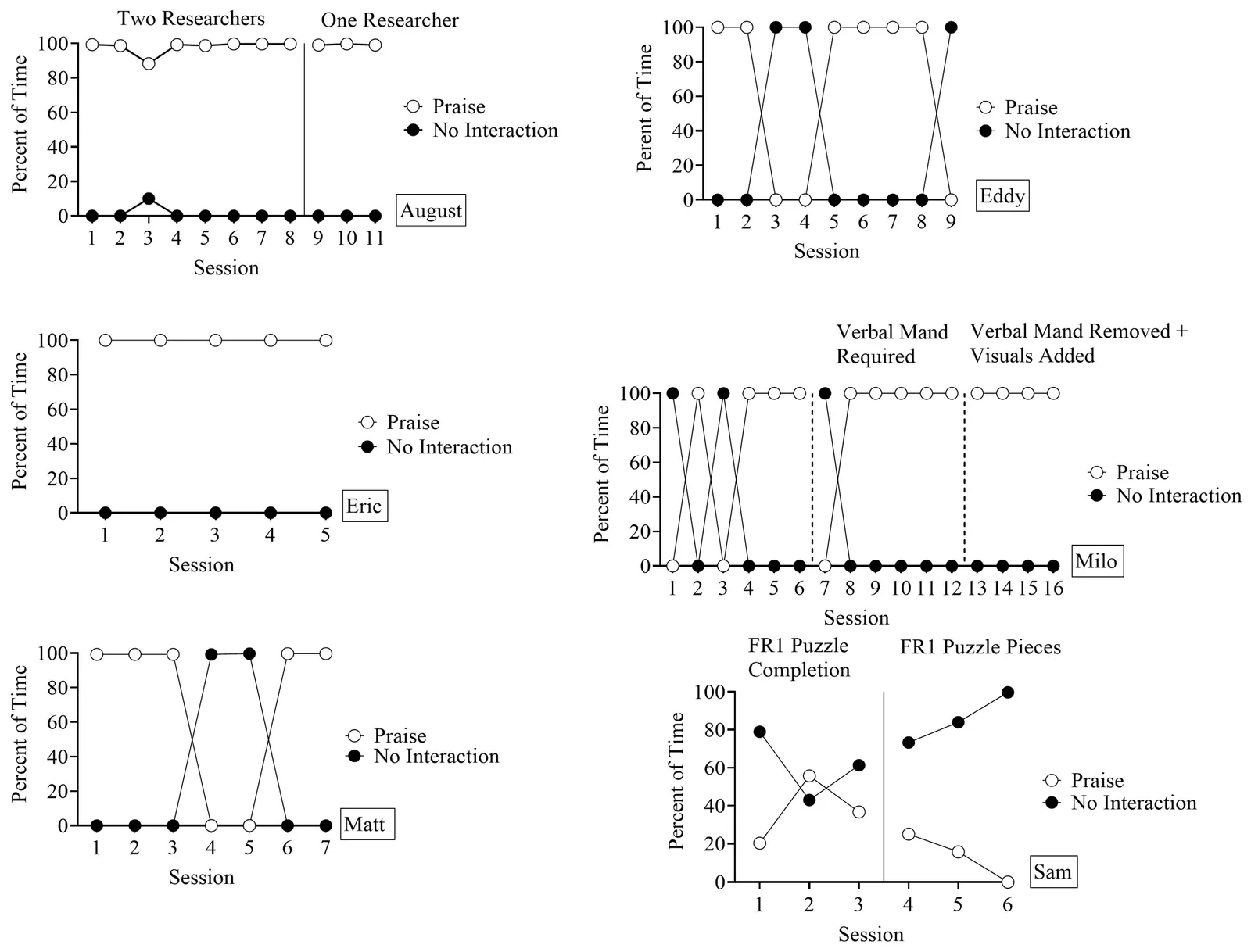
Results for all participants during the COA in the comparison praise vs. no interaction.

Figure 1 displays the percentage of time allocation results for all six participants during the praise vs. no interaction comparison. August and Eric (first column, first and second panel) allocated a majority of their time to the praise side when no interaction was concurrently available. Eddy and Matt (second column, first panel, and first column, third panel) showed variability with their time allocation, but, overall, more time was allocated to the praise side than the no interaction side. For Milo (second column, second panel), there was also variability in responding within the first three sessions. Across all three comparisons, it appeared that Milo demonstrated a side bias for one side of the table. A modification was made where, instead of having Milo choose a side, the lead researcher asked him, “Where do you want to sit?” and he was prompted to provide a verbal response for the consequence he preferred. For example, if asked “Do you want (RA name) to be busy or tell you good job when you do your work?”, he would answer with “busy” or “good job.” Once this modification was made, although the sides of the table where a specific consequence was located was randomized, the session leader’s verbal behavior (e.g., “Do you want (researcher name) to tell you what a good job you are doing when you do your work or do you want her to be busy and not talk to you when you do your work?”) did not alternate. Once this modification was made, there appeared to be a recency bias where Milo would always choose the second-choice option. Therefore, an additional modification was made where the verbal mand requirement was removed, and corresponding visuals were added on both sides of the table depicting either praise, no interaction, or preferred conversation topics. Before each session, a brief training on the receptive identification of the visuals was implemented to ensure Milo was able to discriminate between the visual stimuli. Following the second modification, we observed a higher allocation to the praise side instead of the no interaction side. For participants whose procedures were modified during the assessment, changes in responding following a procedural modification cannot be attributed solely to the programmed social consequence. This limitation should be considered when interpreting a participant’s individual data. For Sam (second column, third panel), we observed undifferentiated responding between the praise and no interaction side. Sam frequently switched sides before completing the puzzle; therefore, her behavior was not coming into contact with the contingencies. Thus, the procedures were modified to deliver the consequence contingent on each puzzle piece being placed in the correct spot instead of full puzzle completion. When the response requirement was modified, there was an increasing trend towards time allocation on the no interaction side when praise was concurrently available.

**Figure 2:**
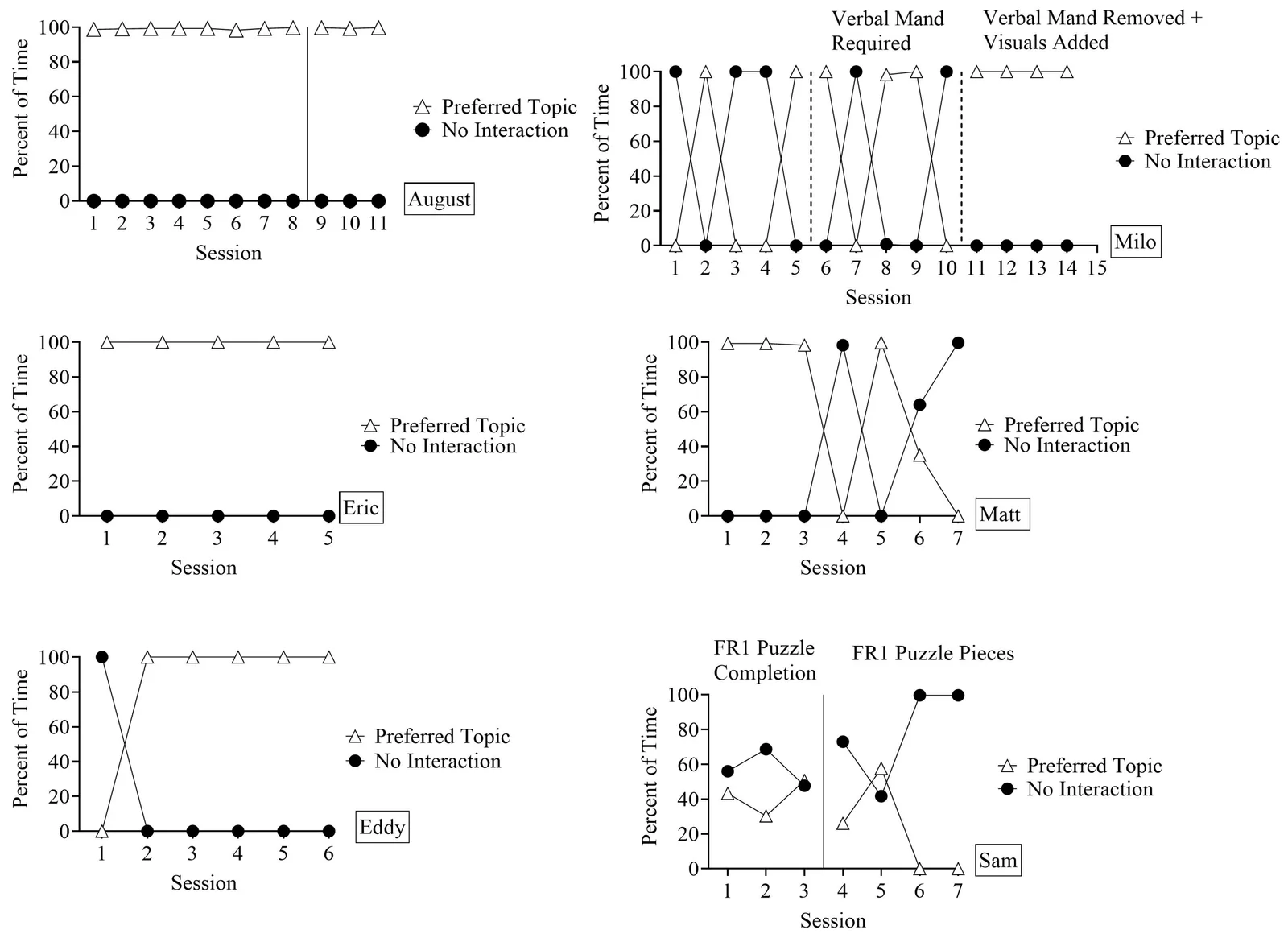
Results for all participants during the COA in the comparison preferred topics vs. no interaction.

Figure 2 displays the percentage of time allocation results for all six participants during the preferred topic vs. no interaction side. August, Eric, and Eddy (first column and first, second, and third panel) demonstrated a higher time allocation for the preferred topic side when no interaction was concurrently available. For Sam (second column, third panel), there was undifferentiated responding in the first phase. When the response requirement modification was made, variability appeared with the first two data points, and then there was an increase in allocation to the no interaction side. Milo (second column, first panel) had variable responding across the first phase and second phase when a verbal mand was required. In the third phase, where the verbal mand requirement was removed, and visuals were added, he allocated more time to the preferred conversation topics side. Matt’s time allocation (second column, second panel) was variable in his allocation to the preferred conversation topic and no interaction side.

**Figure 3:**
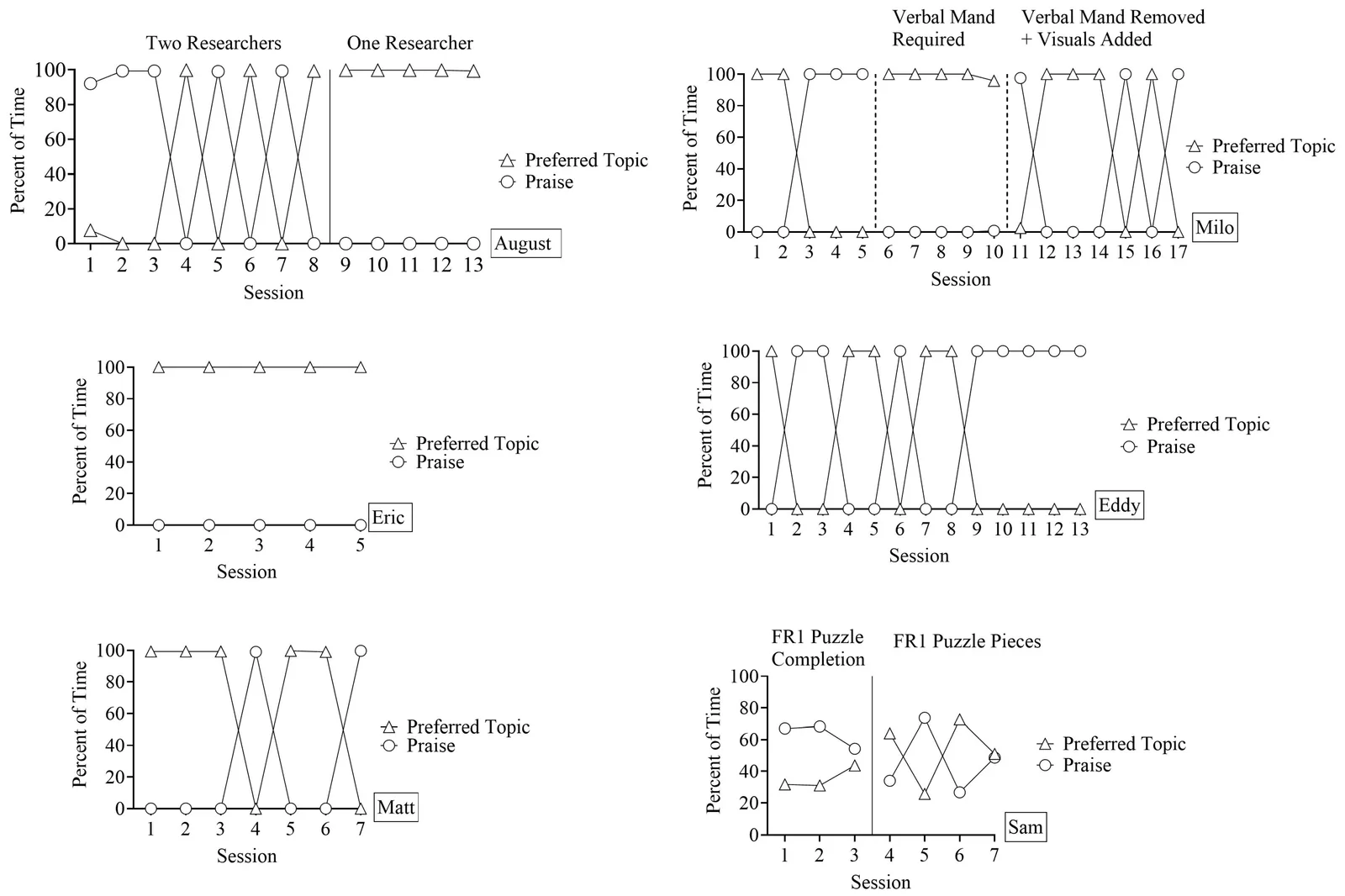
Results for all participants during the COA in the comparison preferred topics vs. praise.

Figure 3 displays the percentage of time allocation results for all six participants during the praise versus preferred conversation topics comparison. Eric (first column, second panel) allocated all of his time to the preferred conversation topics side when praise was concurrently available. For August (first column, first panel), during the first phase with two therapists in the room, he did not consistently allocate his time toward the praise or preferred conversation topics side. In between the sessions, he verbally reported to the researchers who his favorite RA was (thus, it was a preference for a person rather than for the contingencies being tested). Once a change was made in the second phase to only having one RA deliver the consequence, there was an increase in time allocation to the preferred topic side. For Sam (second column, third panel), there was variable time allocation to the praise side. When the response requirement modification was implemented, there was continued variable allocation. For Milo (second column, first panel), during the first phase, we observed slightly more allocation to the praise side than the preferred topic side. During the second phase, where a verbal mand was now required, he consistently allocated his responding to the preferred conversation topics side. During the third phase, when the verbal mand was removed and visuals were added, there was variable allocation observed. For Matt (first column, third panel), there was variability with his time allocation. For Eddy (second column, second panel), there was also variability in time allocation.

Table 4 demonstrates the mean percentage of time allocation across each comparison condition for each participant. During the comparison condition praise versus no interaction, August had a mean rate of time allocation of 98.94% on the praise side and 0.91% on the no interaction side. During the comparison condition preferred topic versus no interaction, August had a mean rate of time allocation of 100% on the preferred topic side and 0% on the no interaction side. During the comparison condition praise versus preferred topic, August had a mean rate of time allocation of 23.67% on the praise side and 76.31% on the preferred topic side. During the comparison condition praise versus no interaction, Sam had a mean rate of time allocation of 25.57% on the praise side and 73.43% on the no interaction side. During the comparison condition preferred topic versus no interaction, Sam had a mean rate of time allocation of 29.72% on the preferred topic side and 69.59% on the no interaction side. During the comparison condition praise versus preferred topic, Sam had a mean rate of time allocation of 57.57% on the praise side and 41.36% on the preferred topic side. During the comparison condition praise versus no interaction, Matt had a mean rate of time allocation of 71.43% on the praise side and 28.57% on the no interaction side. During the comparison condition preferred topic versus no interaction, Matt had a mean rate of time allocation of 62.14% on the preferred topic side and 37.71% on the no interaction side. During the comparison condition praise versus preferred topic, Matt had a mean rate of time allocation of 28.57% on the praise side and 71.43% on the preferred topic side. During the comparison condition praise versus no interaction, Eric had a mean rate of time allocation of 100% on the praise side and 0% on the no interaction side. During the comparison condition preferred topic versus no interaction, Eric had a mean rate of time allocation of 100% on the preferred topic side and 0% on the no interaction side. During the comparison condition praise versus preferred topic, Eric had a mean rate of time allocation of 0% on the praise side and 100% on the preferred topic side. During the comparison condition praise versus no interaction, Milo had a mean rate of time allocation of 81.25% on the praise side and 18.75% on the no interaction side. During the comparison condition preferred topic versus no interaction, Milo had a mean rate of time allocation of 73.22% on the preferred topic side and 26.72% on the no interaction side. During the comparison condition praise versus preferred topic, Milo had a mean rate of time allocation of 33.24% on the praise side and 66.57% on the preferred topic side. During the comparison condition praise versus no interaction, Eddy had a mean rate of time allocation of 66.67% on the praise side and 33.33% on the no interaction side. During the comparison condition preferred topic versus no interaction, Eddy had a mean rate of time allocation of 83.33% on the preferred topic side and 16.67% on the no interaction side. During the comparison condition praise versus preferred topic, Eddy had a mean rate of time allocation of 61.54% on the praise side and 38.46% on the preferred topic side. Table 5 demonstrates, for each participant across each comparison condition, the mean rate of responses completed and the mean cumulative number of responses completed. During the comparison condition praise versus preferred topic, August, Matt, Eric, and Milo had a higher mean rate of task completion and cumulative number of responses while on the preferred conversation topic side. Sam and Eddy had a higher mean rate of task completion on the praise side instead of the preferred topic side. Eddy had a higher number of cumulative responses on the praise side and Sam had a higher number of cumulative responses on the preferred topic side. During the comparison condition praise versus no interaction, August, Matt, Eric, Milo, and Eddy had a higher mean rate of task completion and cumulative number of responses while on the praise side. Sam had a higher rate of task completion and cumulative number of responses on the no interaction side. During the comparison condition preferred conversation topics versus no interaction, August, Eric, and Eddy had a higher rate of task completion and cumulative number of responses while on the preferred topic side. Milo had a higher rate of task completion while on the no interaction side and a higher rate of cumulative number of responses while on the preferred conversation. Sam had a higher rate of task completion and cumulative number of responses on the no interaction side. Matt had an identical mean rate of task completion and cumulative number of responses while on the preferred conversation topic and no interaction side.

### Social Validity Result

The social validity ratings obtained from participants showed that the assessment procedures were perceived to be moderately to highly acceptable (See Table 6). When asked about what they thought about participating in the study, one participant indicated that “they liked it,” three indicated “they really liked it,” and one indicated “it was okay.” When asked what they liked about the study, two participants indicated they liked doing the tasks, one indicated “happy”, one indicated they liked “everything”, one indicated “I don’t know—learning”, and one indicated “talking about different topics that I liked”. None of the participants reported anything that they disliked about the study. Four participants indicated that they would participate in this study again, and one participant indicated “I don’t know, probably”.

Participants were also asked to report their preferred consequence across all comparisons. Their responses were compared to their COA results. Five out of six participants reported that they preferred conversation topics as a consequence compared to no social consequence. All six participants reported that they preferred conversation topics over praise. Five out of six participants reported that they preferred praise over no social consequence. For Matt, Eric, Milo, and August, their verbal report matched the results from each comparison condition of the COA. For Sam and Eddy, their verbal report corresponded with two out of three conditions. Sam expressed a preference for conversation topics over praise; however, her COA results were undifferentiated when evaluated using visual analysis, and, on average, she allocated more time toward the praise side during the comparison condition. Eddy reported a preference for conversation topics over praise. However, he allocated more time toward praise during the COA.

## 4. Discussion

### 4.1. Key Findings

This study served as a proof-of-concept to evaluate a methodology for determining whether individuals with ASD would allocate their time to social consequences such as praise or preferred conversation topics, compared to no social consequence. Overall, the results of this study demonstrated that participants generally allocated their time to the praise or preferred conversation topics side relative to no interaction for engaging in a mastered task. During the praise and no interaction comparison, five out of six participants allocated most of their responding to the praise side, indicating a possible preference for praise. August, Eric, Milo, Matt, and Eddy all demonstrated a higher cumulative number of responses and mean rate of task completion while on the praise side versus the no interaction side. Although Sam demonstrated a higher number of cumulative responses and mean rate of task completion on the no interaction side, it was observed that Sam did allocate more time away from the praise side after the response requirement changed. This may be due to the FR1 schedule and density of praise statements which may be aversive for some individuals who do not prefer high rates of continuous attention. Similarly, it is possible that the delivery of praise may have become aversive for Eric. When he was on the praise side, he would ask, “Why do you keep saying those things?”, which may indicate an aversion to continuous praise statements.

During the preferred conversation topics and no interaction comparison, five out of six participants allocated more time toward listening or talking about preferred conversation topics, indicating a possible preference for conversation as a consequence. August, Eric, Milo, and Eddy had a higher cumulative number of responses and mean rate of tasks completed on the preferred topic side relative to the no interaction side. Sam had a higher number of cumulative responses and mean rate of task completion on the no interaction side. For Matt, he had an equal number of cumulative responses and mean rate of task completion on both the preferred topic and no interaction side. During sessions, we did see variability in participant interactions. For example, it was observed, during some sessions, that Matt would verbalize different statements while on the no interaction side such as “This is so easy!”. It was also observed that Milo would periodically talk to the researchers about his preferred conversation topics and smile at them while on the no interaction side. Evidently, these results may also suggest that some individuals with autism prefer social interactions with others, at least relative to no social interaction.

During the preferred conversation topics and praise comparison, four out of six participants allocated time to preferred conversation topics instead of praise. August, Eric, Milo, and Matt demonstrated a higher cumulative number of responses and mean rate of task completion while on the preferred topic side. Sam demonstrated a higher mean rate of task completion while on the praise side although she had a slightly higher number of cumulative responses on the preferred topic side. Eddy had a higher mean rate of tasks completed while on the praise side and also a higher cumulative number of responses while on the praise side.

During the social validity assessment, we observed a correspondence between the time allocation and verbal report for four out of six participants across each comparison. Sam reported a preference for conversation topics, but this was not demonstrated in the COA. Notably, for Sam, the conversation topics and praise comparison is the only condition where we observed undifferentiated responding. It is possible that Sam simply did not have a preference. We did not include a “no preference” option in the social validity survey; thus, future researchers should consider adding this as an option for participants who may not have a preference. Sam’s sessions were disrupted due to scheduling conflicts, and, thus, we could not continue her evaluation. We also observed a discrepancy in Eddy’s verbal report and COA results. Eddy reported a preference for preferred topics; however, he allocated more time toward the praise side. Prior to the consistent responding in his last five sessions, Eddy’s selections were variable and it was observed that, while he was on the preferred topic side, he did not initiate any conversations with the researcher and also did not reliably respond to questions. It was observed that Eddy would mainly repeat scripts from media or sing songs while completing tasks on all sides.

### 4.2. Limitations and Future Research

Throughout this study, the durations of praise statements versus preferred conversation topics statements/questions were not equated. This presents as a confounding variable for the study as it is possible that participants may have allocated more time to the preferred topic side to access a longer duration of social interaction, greater reciprocal engagement, increased social attention, richer linguistic input, or longer exposure to the reinforcing stimulus. In the current study, we did not equalize the duration of praise statements and preferred topic statements/questions because, for our initial proof-of-concept, we prioritized making the social interactions seem more natural. It is possible that it may have been awkward for a researcher to suddenly stop talking about a preferred topic or only talk about it for a short period of time. It may also have been awkward for the researchers to give praise statements for unusually prolonged periods if the duration of praise and preferred topics were exactly matched. The same could be true for the listener in these cases.

Differences in the task complexity or intrinsic motivation may have influenced the response allocation independently of the programmed consequences. Intrinsic motivation is observed when individuals engage in an activity because they experience interest and enjoyment and not because of an extrinsic reward (Deci, 1975). It is possible that engaging in a task itself may have been reinforcing for some participants regardless of the consequence. Additionally, based on the researchers’ descriptive observations, participants’ communication skills may have influenced preference. Eric, Sam, and Matt had advanced communication skills, although their data portrayed variable time allocation across comparisons. August, Milo, and Eddy had the least advanced communication skills; however, they allocated more time towards preferred topics over praise or no social interaction. Although Eddy did not seem to engage in conversation with the researchers, Milo and August did attempt to have back and forth interactions. It is important to note that participants with limited conversation skills, such as Milo and August, appeared to enjoy conversations with the researchers related to their preferred topics. It is possible that, for individuals like Milo and August, clinicians may be able to build upon conversation skills by using preferred topics. Future researchers may explicitly explore the relation between communication ability and preference for social interaction. During this study, low IOA was reported for Milo for task completion. IOA was completed for less than 33% of sessions due to the difficulties observing task completion within the recorded videos. Because of the camera angle, two observers had difficulty agreeing on how many responses occurred. In the future, researchers should aim to collect in vivo IOA or continuously monitor IOA while the study is in progress.

Due to the variability in responding across participants, several procedural adaptations were made. Sam, and Matt experienced the COA with two adults providing consequences whereas Eddy, Milo, and Eric experienced the COA with one adult delivering both consequences. August experienced both of these procedures. These changes were made to mitigate any potential bias towards a specific researcher which may have introduced a confounding variable. In the first phase (FR1 puzzle completion), Sam would only complete a full puzzle once or twice; thus, she rarely contacted the predetermined consequences during sessions. Because of this, the response requirement was changed from completing a whole puzzle to receiving the consequence for every one puzzle piece placed, to increase her exposure to the consequences. Milo experienced adaptations such as adding a verbal response requirement during the COA and adding corresponding visual cues to both sides of the table due to side and recency bias. Eric and Milo also worked on two different mastered tasks during the sessions due to them completing the tasks very quickly and, therefore, it was difficult to provide the consequences on an FR1 schedule. Milo was also observed having difficulty with the matching task. The physical arrangement of materials such as the mastered task also differed across participants. August, Sam, Matt, and Eric all had two identical tasks placed on both sides of the table and Milo and Eddy had one task placed on the middle of the table. Milo and Eddy had this adaptation because it was observed that previous participants would take the task they were working on previously to the side that they chose. Participants were also observed flipping pages in the worksheet packets seemingly to avoid redoing any pages that they had already completed while seated at a different side of the table. Due to these procedural modification participants were not exposed to equivalent procedures which limits cross-participant comparison. Although these adaptations were clinically appropriate and necessary to reduce bias in responding and ensure contingencies were met, their cumulative effect introduced procedural heterogeneity that limits internal validity. Because participants were no longer exposed to equivalent experimental conditions, it becomes difficult to determine whether observed differences reflect genuine preferences for social consequences or are partly attributable to procedural modifications. To address these limitations, we are currently developing a revised iteration of the study in which procedural modifications will be limited and the duration of praise and preferred conversation statements will be equalized.

During this study we did not assess individual preference for conversation style. Preferred conversation topics were delivered as a statement or a question relating to the participants’ most preferred conversation topics. We did not equate the number of statements versus questions that the researchers delivered (during the preferred topic side). It is unknown whether participants who allocated time to the no interaction side or praise side instead of the preferred topic side, such as Matt, Eddy, and Sam, may have found questions or statements as less (or more) preferred. Although it was not required for participants to respond to researcher-initiated verbal behavior, we observed that not all participants engaged in conversations with the researchers or responded to them. We observed variability in conversational behavior, such as how some participants would complete one task and then initiate a conversation themselves or mand for the researcher to engage in their preferred conversation topics. For example, Eric would engage in one response and say “Tell me a scary story” while continuously working on his task. Other participants, such as August or Milo, would complete one task and look up at the researcher while waiting for them to initiate a statement or question. We observed that Eddy would work on his task and occasionally engage in singing or repeating scripts from media; thus, it was unclear if he was initiating a conversation with the researcher. We also observed variability with participant verbal behavior relating to providing on-topic statements, off-topic statements, on-topic questions, and off-topic questions while they were on the preferred topic side. One variable that may have influenced time allocation to the no interaction, praise, or preferred topic side are participant’s communication or conversational skills. Perhaps individuals with ASD who have more advanced communication skills would choose to allocate their time to the preferred topic side rather than the praise or no interaction side due to their prior learning history. Future researchers should consider evaluating preference for conversation style. For example, researchers may want to assess whether individuals with autism prefer to receive statements or questions or both about their preferred conversation topics when speaking to another person. An additional assessment may help to further assess using conversation topics as a consequence for task completion (or other responses).

Another limitation of the study is that we did not assess which features of the verbal interaction were reinforcing for participants who allocated more time to the preferred topic side. For example, it is unknown if participants enjoyed listening to the researcher talk about their preferred conversation topics, if they preferred talking about their preferred conversation topics, if they preferred other features of the interaction (including verbal and nonverbal components), or some combination. Throughout the study, some participants engaged in a typical back-and-forth conversation while other participants engaged in more repetitive verbal behavior. For example, Milo frequently asked the researchers questions related to their schedule such as “What time did you wake up today?” Additionally, Eddy often would not respond to statements or questions initiated by the RAs. Future studies could parse out features of a social interaction to identify the reinforcing components. This information may aid with learning how to enrich conversations as a reinforcing experience for individuals with ASD. Additionally, the current study contained a small sample size (N = 6). The study results should be viewed as preliminary. In the future, researchers should recruit a larger sample size for further empirical support of the study procedures.

The present findings open several avenues for future research that can extend and refine the methods. One additional area involves attention satiation and habituation related to repeatedly discussing or hearing the same preferred conversation topic (or praise statement, for that matter). Future researchers should also examine whether preferred conversation topics can reinforce task behavior when embedded within other behavioral procedures, such as progressive ratio schedules. Finally, future research should evaluate the feasibility and effectiveness of individualized social reinforcers, including preferred conversation topics, across a broader range of settings such as classroom, vocational, and workplace environments.

## 5. Conclusions

The findings of this study further support that COAs can be an effective method for identifying individual preferences for different forms of social interaction for individuals with ASD. Behavior analytic literature has limited use for incorporating a child’s preferred conversation topics within preference assessments in research and clinical settings. The results of this study show that individuals with ASD may prefer to talk about, or, at least, hear someone talk about, their preferred conversation topics. At a minimum, the study shows the viability of an assessment to evaluate preferences for social interaction. However, the ecological validity of these findings is not yet known. It is not known whether the preferences observed in this study would be similar in natural environments such as school, home, or community settings.

## Figures and Tables

**Table 1:** Social validity interview form.

Question	Response Options
1. How do you feel about participating in this study? * How would you rate the tasks that you completed in the study?	I really didn’t like it, I did not like it, it was okay, I liked it, I really liked it * 1 = extremely dislike, 7 = extremely like
2. What did you like about the study if anything?	Open ended
3. What did you dislike about the study, if anything?	Open ended
4. Did you like it better when we said you were doing a good job or when we were busy and did not talk to you?	Open ended
5. Did you like it better when we talked to you about your favorite things (insert preferred conversation topics) or when we were busy and did not talk to you?	Open ended
6. Did you like it better when we said you were doing a good job or when we talked to you about your favorite things (insert preferred conversation topics)	Open ended
7. Would you do this study again? * How likely are you to participate in this study again?	Yes, Maybe, No * 1 = very unlikely, 7 = very likely

Note. * Indicates questions and response options delivered to August.

**Table 2:** Hierarchy of topic preference produced by MSWO preference assessments.

Participant	MSWO
August	VHS Tapes
	Boba Tea
	Making Friends
	Video Editing
	Sports
	Classic TV Shows
Sam	Reptiles & Amphibians
	Track & Field
	Roblox
	Murder Mysteries
Matt	Minecraft
	Theme Parks
	Monster Trucks
	Food
Eric	Scary Stories
	Video Games
	Drawing
	School Games
	Books
Milo	Disney World
	Mario
	Little Einsteins
	Schedules
	Roller Coasters
Eddy	Movies and Books
	Commercials
	Cars
	Songs/Singing
	Drawing

**Table 3:** Duration of praise or preferred conversation topics delivered by the researcher.

Participant	Duration of Praise	Duration of Preferred Conversation Topics
August	3.8 s	8.9 s
Eric	2.3 s	5.3 s
Milo	2.0 s	3.6 s
Eddy	2.4 s	4.5 s

**Table 4:** Mean percentage time allocation per condition per participant.

	Praise v NI		PT v NI		Praise v PT	
Participant	Praise	NI	PT	NI	Praise	PT
August	98.94	0.91	100	0	23.67	76.31
Sam	25.57	73.43	29.72	69.59	57.57	41.36
Matt	71.43	28.57	62.14	37.71	28.57	71.43
Eric	100	0	100	0	0	100
Milo	81.25	18.75	73.22	26.72	33.24	66.57
Eddy	66.67	33.33	83.33	16.67	61.54	38.46

**Table 5:** Mean rate and cumulative responses (tasks completed) for each participant across each comparison condition.

		Praise v PT		Praise v NI		PT v NI	
Participant		Praise	PT	Praise	NI	PT	NI
August	Mean Rate	0.81	1.08	2.18	0.02	1.41	0
	Cumulative Responses	49	70	120	1	78	0
Sam	Mean Rate	0.31	0.28	0.2	0.36	0.17	0.48
	Cumulative Responses	10	11	6	11	6	16
Matt	Mean Rate	0.22	0.46	0.34	0.17	0.26	0.26
	Cumulative Responses	8	16	12	6	9	9
Eric	Mean Rate	0	2.8	4	0	4	0
	Cumulative Responses	0	28	53	0	40	0
Milo	Mean Rate	7.91	9.95	14.27	7.33	7.36	10.62
	Cumulative Responses	114	229	371	44	162	85
Eddy	Mean Rate	1.61	0.81	1.67	1	1.83	0.42
	Cumulative Responses	42	21	30	18	22	5

Key: PT—Preferred Topics, NI—No Interaction.

**Table 6:** Participant’s response to social validity questionnaire.

Question	Matt	Sam	Eric	Milo	Eddy	August
How do you feel about participating in this study? * How did you feel about the task (insert task) you completed in this study?	It was okay	I liked it	I really liked it	I really liked it	I really liked it	* 5
What did you like about the study if anything?	“The puzzles were fun”	“I liked to do puzzles”	“Everything”	“Happy”	“I don’t know—learning”	“Talking about different topics that I liked”
What did you dislike about the study, if anything?	“Nothing”	“Nothing”	“Nothing”	(Did not provide an answer)	(Did not provide an answer)	“Nothing”
Did you like it better when we said you were doing a good job or when we were busy and did not talk to you?	“Good job”	“Didn’t talk”	“Good job”	“Good job”	“Good job”	“Good job”
Did you like it better when we talked to you about your favorite things (insert preferred conversation topics) or when we were busy and did not talk to you?	“Favorite things”	“Didn’t talk”	“Favorite things”	“Favorite things”	“Favorite things”	“Favorite things”
Did you like it better when we said you were doing a good job or when we talked to you about your favorite things (insert preferred conversation topics)	“Favorite things”	“Favorite things”	“Favorite things”	“Favorite things”	“Favorite things”	“Favorite things”
Would you do this study again?	“I don’t know—probably”	Yes	Yes	Yes	Yes	* 5

* August’s response to the first and last questions based on a Likert-scale with the rating of 1 to 5.

## Data Availability

The data presented in this study are available upon request from the corresponding author. The data are not publicly available due to privacy restrictions.

## Supplementary Materials

The following supporting information can be downloaded at: https://www.mdpi.com/article/10.3390/bs16091695/s1, Table S1: MSWO Implementation Procedural Integrity Scores; Table S2: Pre Exposure Script Implementation Procedural Integrity Scores; Table S3: Researcher Delivery of Praise, Preferred Topics, or No Interaction Procedural Integrity Scores.
