# Supplementary material for: Assessing Social Consequences for Task Completion in Individuals with Autism Spectrum Disorder
Source: Behav Sci (Basel). 2026 Sep 20;16(9):1695. doi: 10.3390/bs16091695 (PMC13603383; doi:10.3390/bs16091695)
Supplement: Supplementary file 1 [file behavsci-16-01695-s001.zip › behavsci-4430370-supplementary.pdf]

Supporting Information

Table S1

MSWO Implementation Procedural Integrity Scores

Participant Name	Percent of Sessions with Procedural Integrity	Mean Procedural Integrity
Eddy	66%	93.3%
Eric	Not Available	Not Available
August	66%	100%
Milo	12%	100%
Matt	Not Available	Not Available
Sam	Not Available	Not Available

Table S2

Pre Exposure Script Implementation Procedural Integrity Scores

Participant Name	Percent of Sessions with Procedural Integrity	Mean Procedural Integrity
Eddy	75%	90%
Eric	13%	80%
August	48%	90%
Milo	89%	91%
Matt	71%	100%
Sam	70%	100%

Table S3

Researcher Delivery of Praise, Preferred Topics, or No Interaction Procedural Integrity Scores

Participant Name	Percent of Sessions with Procedural Integrity	Mean Procedural Integrity
Eddy	75%	100%
Eric	93%	100%
August	66%	100%
Milo	52%	100%
Matt	43%	100%
Sam	25%	100%
